# Serum-free light chain test utilisation at a South African academic laboratory and comparison with serum protein electrophoresis results

**DOI:** 10.4102/ajlm.v12i1.2201

**Published:** 2023-11-24

**Authors:** Razia B. Banderker, Fatima B. Fazel, Annalise E. Zemlin, Aye-Aye Khine, Thumeka P. Jalavu

**Affiliations:** 1 Department of Pathology, Division of Chemical Pathology, Faculty of Medicine and Health Sciences, Stellenbosch University, Cape Town, South Africa; 2 Department of Pathology, Division of Chemical Pathology, National Health Laboratory Service, Cape Town, South Africa; 3 Department of Internal Medicine, Division of Clinical Haematology, Faculty of Medicine and Health Sciences, Stellenbosch University, Cape Town, South Africa; 4 Division of Chemical Pathology, National Health Laboratory Service, Green Point, Cape Town, South Africa

**Keywords:** monoclonal gammopathy, multiple myeloma, M-protein, paraprotein, plasma cell dyscrasia, polyclonal gammopathy, free light chains

## Abstract

**Background:**

Serum protein electrophoresis (SPE), urine protein electrophoresis and immunofixation electrophoresis were traditionally utilised for the diagnosis of monoclonal gammopathies. The quantitative serum-free light chain (SFLC) assay is reportedly more sensitive and has been introduced to recent clinical guidelines.

**Objective:**

This study aimed to investigate SFLC test utilisation and describe SPE findings in patients with abnormal SFLC ratios.

**Methods:**

A retrospective audit of SFLC analyses was conducted in Cape Town, South Africa, from May 2018 to April 2020. Agreement between abnormal SFLC ratios and SPE results was determined in a sub-group of patients screened for monoclonal gammopathies. Serum-free light chains were analysed using Freelite^®^ Kappa and Lambda assays.

**Results:**

Of the 1425 patients included in the audit, 741 (52%) had abnormal SFLC ratios; 636 (45%) had increased and 105 (7%) had decreased SFLC ratios. In a sub-group analysis of 117 new patients with an abnormal SFLC ratio, 57 had a monoclonal protein (M-protein) on SPE (49%), and 60 (51%) did not. Four out of 60 patients without M-protein had a plasma cell dyscrasia, while renal impairment or inflammatory response accounted for the rest. Of the 57 patients with a M-protein and abnormal SFLC ratio, 41 (72%) had a plasma cell dyscrasia, seven (12%) had lymphomas and nine patients (16%) were unclassifiable.

**Conclusion:**

Serum-free light chains should be requested when there is a high index of clinical suspicion. Neither SFLC nor SPE should be performed in isolation when screening patients for monoclonal gammopathy, to ensure that no patient is missed.

**What this study adds:**

The study adds to the evidence on SFLC test utilisation. Serum protein electrophoresis alone may miss cases of light chain myeloma, while SFLC performed in isolation may produce false positive results in the setting of inflammatory disorders or renal impairment, leading to unnecessary further investigation.

## Introduction

Monoclonal gammopathies (MG) comprise a spectrum of disorders, from the pre-malignant Monoclonal Gammopathy of Undetermined Significance (MGUS) to Multiple Myeloma (MM), which is associated with significant morbidity and mortality.^1^ A hallmark of MG is the production of a monoclonal protein (M-protein) (except in cases of non-secretory MM).^2^ Some synonyms include ‘paraprotein’, ‘M-component’, and ‘monoclonal immunoglobulin’. The M-protein may consist of intact immunoglobulins (heavy and light chains bound together), free light chains (not bound to a heavy chain) or fragments of either one. The M-protein is produced more commonly by a malignant clone of plasma cells but may also be produced by clonal lymphoplasmacytic cells (as seen in Waldenström’s macroglobulinaemia).^3^ According to the 2019 Cancer Association of South Africa Cancer Registry statistics, MM accounted for 0.5% of all histologically diagnosed cancers. The most common age group at diagnosis was 60–64 years among both male and female patients.^4^

For decades, serum protein electrophoresis (SPE) and urine protein electrophoresis (UPE) and immunofixation electrophoresis (IFE) were the initial tests for diagnosing MG. A limitation of SPE is that it can only identify M-proteins with a concentration greater than 500 mg/L – 2000 mg/L, and patients with lower concentrations of M-protein, such as those with oligosecretory myeloma, may be missed, especially if the M-protein is a free light chain.^5,6^ Also, for accurate quantification of the M-protein in urine, a 24-h urine collection is preferred over a random specimen. Collection of urine for 24 h is inconvenient for patients and often incorrectly collected. However, a recent local study suggested random urine specimens may be used to estimate the 24-h urine M-protein concentration with equations in patients previously diagnosed with MM.^7^ The quantitative serum-free light chain (SFLC) assay was developed in 2001 by The Binding Site Group^5^ and can detect light chains at concentrations less than 1 mg/L, making it a more sensitive method than SPE and UPE.^8^ The International Myeloma Working Group released a consensus guideline for investigating patients suspected of having MM in 2011^9^ and guidelines on the use of The Binding Site SFLC assay in screening, diagnosis and monitoring of MG in 2009.^10^ The two recommended screening profiles are either SPE with IFE and the SFLC assay, or SPE with IFE and UPE with urine immunofixation electrophoresis (UIFE).^11^

The SFLC assay was added to our laboratory test repertoire in 2018. Although SPE and SFLC have been recommended, we have subjectively noted that clinicians at our centre frequently request a single test (either SPE or SFLC) when screening patients for MG. This practice may be an attempt to reduce hospital expenditure; however, it may lead to false-negative screening results and poorer patient outcomes. This study aimed to investigate SFLC test utilisation and to describe the SPE findings in those with abnormal SFLC ratios in the screening of patients for MG at Tygerberg Hospital (TBH) National Health Laboratory Service (NHLS).

## Methods

### Ethical considerations

This study was approved by the Health Research Ethics Committee of Stellenbosch University (S19/10/270, 05 December 2019) and was performed according to the Declaration of Helsinki. Informed consent was not required as the study was a laboratory-based, retrospective audit. All results were de-identified to maintain patient confidentiality and only shared with members of the study team when deemed necessary. Data were stored on password-protected devices.

### Study design

The study was a retrospective audit of laboratory data and clinical folders of select adult patients on whom SFLC assays were requested at TBH NHLS for 2 years, from May 2018 to April 2020.

### Setting

Tygerberg Hospital is a 1380-bed tertiary academic hospital in Cape Town, Western Cape, South Africa, affiliated with Stellenbosch University’s Faculty of Medicine and Health Sciences. The NHLS is the sole laboratory service provider for public healthcare in South Africa. The NHLS laboratory at TBH participates in internal and external quality control programmes and is an International Organization for Standardization 15189 accredited laboratory. Serum-free light chain samples are referred for analysis from regional hospitals and clinics surrounding TBH.

### Data collection

**FIGURE 1:**
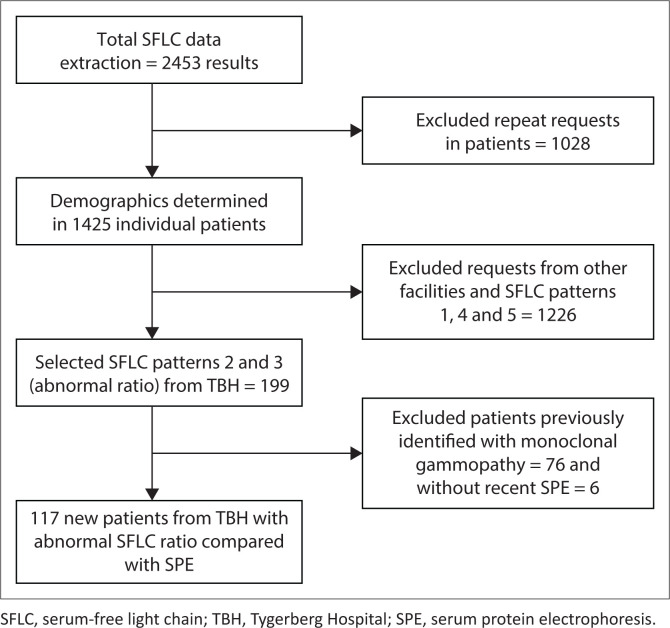
Stepwise exclusion of laboratory data in the study conducted at Tygerberg Hospital National Health Laboratory Service, South Africa, from May 2018 to April 2020.

Data of all SFLC performed in the above period were extracted and anonymised from the NHLS Laboratory Information System (TrakCare Lab Enterprise, InterSystems Corporation, Cambridge, Massachusetts, United States) by the Corporate Data Warehouse. Serum-free light chain results were classified into five groups: 1 – normal kappa (κ) and lambda (λ) free light chains with normal κ/λ ratio, 2 – raised κ/λ ratio, 3 – decreased κ/λ ratio, 4 – raised κ and λ with normal κ/λ ratio (inflammatory pattern), and 5 – other (normal κ/λ ratio with raised or decreased κ or λ free light chains). Stepwise exclusions were performed (Figure 1). Repeat SFLC requests in individual patients were excluded as patients with a MG would have multiple requests over time, skewing demographic statistics. After the initial demographic analysis, groups 1, 4 and 5, and all results from sites other than TBH, were excluded to allow comparison of abnormal SFLC ratio with electrophoresis results. Results of TBH patients were selected as the electronic medical records were accessible for these patients. Medical records of patients seen at other facilities were not readily available. In the final step, patients known with MG and those without an SPE performed within 3 months (90 days) of SFLC testing were excluded. The relationship between abnormal κ/λ ratio and SPE was investigated in TBH patients.

### Laboratory analyses

Serum-free light chain serum samples were analysed using the immunoturbidimetric Freelite^®^ Kappa and Lambda assays (The Binding Site Group Ltd, Birmingham, United Kingdom) on the Roche cobas^®^ 6000 c601 instrument (Roche Diagnostics GmbH, Mannheim, Germany). The coefficient of variation for the Freelite^®^ normal internal quality controls was κ 3.98% and λ 3.79%, and κ 2.52% and λ 4.14% for the high internal quality controls (May 2019). The manufacturer’s reference intervals were previously verified in a local study^12^ and applied as follows: κ free light chain (FLC): 3.3 mg/L – 19.4 mg/L, λ FLC: 5.7 mg/L ‒ 26.3 mg/L, and κ/λ ratio: 0.26‒1.65. Both increased and decreased ratios were considered abnormal.^5^ The reference interval of 0.37‒3.1 for κ/λ ratio in renal impairment was applied in a select group of patients with abnormal ratio and undetectable M-protein on SPE.^13^ Serum protein electrophoresis and IFE were performed on the Sebia Hydrasys 2 (Sebia, Lisses, France) semi-automated agarose gel electrophoresis system. Clinicians at our centre requested SPE, while IFE was reflexively added by the reporting pathologist if necessary, based on the clinical context or SPE appearance (presence of suspected M-protein or hypogammaglobulinaemia).

### Data analysis

Laboratory data were captured in Microsoft Excel spreadsheets (Microsoft Corporation, Redmond, Washington, United States). Demographic statistics of SFLC requests and the frequency of abnormal SFLC results were determined using Statistical Package for Social Scieces, version 27 (IBM Corp., Armonk, New York, United States). Descriptive statistics were determined, where appropriate.

## Results

**FIGURE 2:**
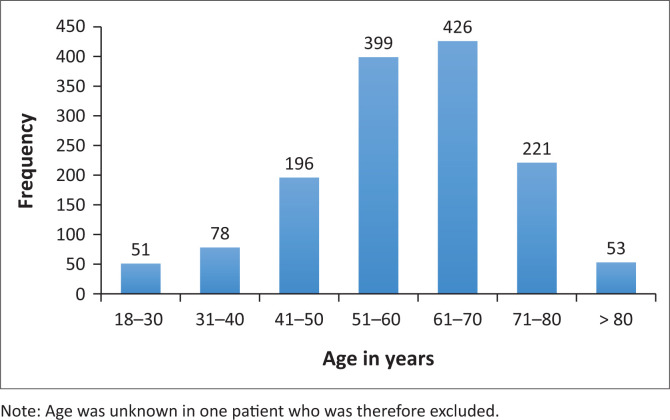
Age distribution of patients for whom serum-free light chain assays were requested at Tygerberg Hospital National Health Laboratory Services, South Africa, from May 2018 to April 2020.

After the data extraction was performed, 1425 individual test results were identified (Figure 1) and included in the retrospective audit. Sixty percent (*n* = 860/1425) of patients were female, with a mean age of 59 years (standard deviation ± 13) (Table 1). The most requests were received for patients in the 51–70-year age group (Figure 2). A minority of requests were received from primary (*n* = 19, 1.3%) and secondary healthcare institutions (*n* = 228, 16%). Most requests were received from tertiary healthcare institutions (*n* = 1178; 82.7%). Just over half (*n* = 741, 52%) of κ/λ ratios were abnormal; 44.6% (*n* = 636) had an increased ratio, and 7.4% (*n* = 105) had a decreased ratio.

**TABLE 1:** Demographics of 1425 patients with serum-free light chain requests received at Tygerberg Hospital National Health Laboratory Services, South Africa, from May 2018 to April 2020.

Demographic characteristics	Frequency	
	Number	%
**Gender**		
Female	860	60.4
Male	565	39.6
**Age in years**		
≤ 40	129	9.1
41–60	595	41.8
> 60	700	49.1
Unknown	1	0.1
**Requesting facility**		
Primary	19	1.3
Secondary	228	16.0
Tertiary	1178	82.7
**Free light chain pattern**		
1 – normal free light chains and ratio	144	10.1
2 – raised ratio	636	44.6
3 – decreased ratio	105	7.4
4 – raised κ and λ with normal ratio (Inflammatory)	377	26.5
5 – other (normal ratio and raised/decreased κ or λ)	163	11.4

Six patients out of 199 (3%) were excluded due to the absence of an SPE within 3 months of the SFLC request. After stepwise exclusion was performed (Figure 1), 117 new patients from TBH with SFLC patterns 2 and 3 (abnormal κ/λ ratio) were identified and had SFLC findings compared with SPE/IFE. Of those, 57 (49%) had an M-protein present on SPE/IFE, and 60 (51%) did not. Forty-one patients were diagnosed with a plasma cell dyscrasia (72%), seven patients had lymphoma (12%), and nine patients were unable to be classified due to the absence of a bone marrow biopsy and incomplete investigation (16%) (Table 2). Out of 28 patients diagnosed with MM, 15 were female, with a female-to-male ratio of 1.2:1. Of the four patients who were HIV-positive, two had MM, one had MGUS, and one had plasmablastic lymphoma.

**TABLE 2:** Clinical features of 57 patients identified with monoclonal gammopathy at Tygerberg Hospital, South Africa, between May 2018 and April 2020.

Clinical characteristics	Number	%
**Age (years)**		
≤ 40	3	5.3
41–60	23	40.3
> 60	31	54.4
**Gender**		
Female	31	54.4
Male	26	45.6
**Diagnoses**		
Plasma cell dyscrasia		
Multiple myeloma	28	49.0
MGUS	8	14.0
Smouldering myeloma	1	1.8
Plasmacytoma	1	1.8
LC deposition disease†	1	1.8
Myeloma cast nephropathy†	2	3.5
Lymphoma		
Waldenström’s macroglobulinaemia	2	3.5
Chronic lymphocytic leukaemia	2	3.5
Diffuse large B-cell lymphoma	1	1.8
Plasmablastic lymphoma	1	1.8
B-cell lymphoma (unspecified)	1	1.8
Incomplete work-up	9	15.7
**HIV status**		
Negative	41	71.9
Positive	4	7.0
Unknown	12	21.1

MGUS, Monoclonal Gammopathy of Undetermined Significance; LC, light chain.

† , Patients diagnosed on renal biopsy. No bone marrow biopsy was performed.

**FIGURE 3:**
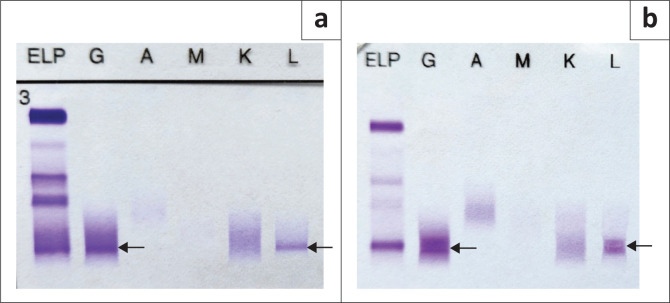
Serum immunofixation gels in two patients with raised κ/λ ratios and λ monoclonal proteins at Tygerberg Hospital, South Africa, between May 2018 and April 2020. a, Patient 1. b, Patient 2. The black arrows indicate positions of the monoclonal proteins in each immunofixation gel.

**FIGURE 4:**
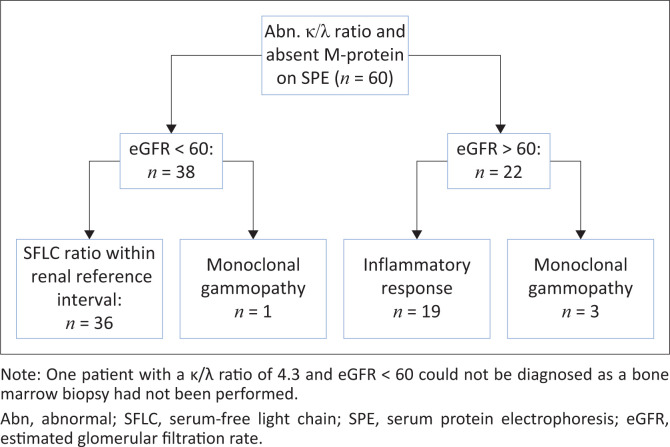
Classification of patients with abnormal ratio and absent M-protein on serum protein electrophoresis at Tygerberg Hospital, South Africa, from May 2018 to April 2020.

Of the 57 patients with MG, two had an immunoglobulin G λ M-protein with an unexpectedly raised κ/λ ratio (Figure 3). Both cases had mildly increased ratios of 1.99 (patient 1, Figure 3a) and 2.09 (patient 2, Figure 3b), respectively (reference interval: 0.26–1.65). Renal impairment could not account for the mildly raised ratios as both patients had normal renal function. Bone marrow biopsy revealed a diagnosis of MM with λ restriction in patient 2, while bone marrow findings were normal in patient 1, suggestive of MGUS. Sixty patients had an abnormal κ/λ ratio without detectable M-protein on SPE and their diagnoses were investigated (Figure 4). In 38 patients with renal impairment, 2 patients (5.3%) had κ/λ ratios outside of the renal κ/λ ratio reference interval (0.37‒3.1). One patient had a κ/λ ratio of 0.13 and was diagnosed with a MG, while the other had a κ/λ ratio of 4.3 but the diagnosis could not be determined as bone marrow biopsy had not been performed. Four patients out of 60 had been diagnosed with a MG (6.7%) based on bone marrow or tissue biopsy findings (Table 3). Of the 19 patients with a presumed inflammatory response, 17 had polyclonal hypergammaglobulinaemia on SPE (89.5%). Most of these were from specialities within internal medicine, namely pulmonology (*n* = 4), neurology (*n* = 4), rheumatology (*n* = 2) and gastroenterology (*n* = 2).

**TABLE 3:** Findings in four patients diagnosed with monoclonal gammopathies, without detectable M-protein on immunofixation electrophoresis at Tygerberg Hospital, South Africa, from May 2018 to April 2020.

Patient number	Creatinine (µmol/L)	eGFR (mL/min per 1.73 m^2^)	Kappa FLC(mg/L)	Lambda FLC (mg/L)	κ/λ ratio	UPE/UIFE	Diagnosis
1	74	> 60	4584.0	9.7	472.58	Free kappa	Light-chain multiple myeloma
2	44	> 60	33.8	10.8	3.13	Negative UIFE	Light-chain multiple myeloma
3	79	> 60	14.2	6.9	2.06	UPE negative (no UIFE)	Plasmacytoma
4	91	56	88.5	705.0	0.13	Not done	Light-chain multiple myeloma

eGFR, estimated glomerular filtration rate; FLC, free light chain; UPE, urine protein electrophoresis; UIFE, urine immunofixation electrophoresis.

## Discussion

The study is one of the first African studies investigating SFLC test utilisation and correlating abnormal SFLC ratios with SPE findings. We found that most patients who underwent MG screening were over 50 and were primarily female. Most SFLC requests came from tertiary healthcare institutions. The most frequently observed SFLC pattern was that of a raised κ/λ ratio. Few patients were excluded for lack of a recent SPE, suggesting that most patients were screened with both SPE and SFLC, with good adherence to clinical guidelines. In the sub-group analysis of patients with abnormal κ/λ ratio, over half did not have a detectable M-protein on SPE. A minority of patients in the sub-group diagnosed with MG were HIV-positive.

As MGs are more frequently suspected and diagnosed in older populations, our finding of more frequent screening in older populations is expected.^14^ The gender distribution in our study was unexpected as higher incidences of MG have been reported internationally among male patients.^15^ A previous study performed at a tertiary hospital in the Western Cape included 100 myeloma patients diagnosed between 2008 and 2015. The authors found a female predominance, with a female-to-male ratio of 1.78:1 (unpublished data). An older study performed at a tertiary hospital in Gauteng (2004–2009), which included 34 myeloma patients, also found a female predominance (2.4:1).^16^ A possible reason for this discrepancy could be a difference in healthcare-seeking behaviour among genders in our population. National census data from 2011 obtained by Statistics South Africa reported that 24.4% of men (compared to 21.1% of women) did not consult a health worker during recent episodes of illness or injury.^17^ This poor healthcare-seeking behaviour among men could also explain why more female patients than male patients were diagnosed with MG in our study cohort. The low number of SFLC requests from primary and secondary healthcare institutions is concerning as many patients could present to their local clinics for non-specific symptoms of MM, such as fatigue or back pain.^14^ A lack of awareness about MG among health workers at the primary care-level facilities may explain the low screening rate. Alternatively, these facilities may not have the budget to request the relatively expensive tests required for screening, necessitating patient referral for further investigation. The relatively few requests received from primary and secondary healthcare institutions may also explain why patients in our setting often present late and with overt signs of end-organ damage.

Many patients newly diagnosed with MG were not thoroughly investigated as they did not have a bone marrow biopsy performed. One patient did not return for the scheduled biopsy, and one had demised before the biopsy could be performed. It is unclear why the rest were not investigated further, although possible reasons could be that the patients demised at home, clinicians did not view or understand the implications of the screening results, or the patients did not return for other reasons (lack of funds to pay for transport or misunderstanding their diagnosis). Additionally, clinicians may have made a preliminary diagnosis of low-risk MGUS in certain patients, thus deferring bone marrow biopsy.^18^ In an audit of IFE and SFLC results between January 2018 and May 2019 performed in India, authors also noted poor follow-up of patients which they attributed to financial constraints, ignorance or patient death.^19^

An unexpected finding in our study was the presence of a raised κ/λ ratio and λ M-protein on SPE in two patients. With a λ M-protein, the ratio should be decreased.^8^ Patient 1’s serum was sent to another laboratory for repeat SFLC testing, and a similar pattern was seen, confirming the result. Patient 2’s serum had not been referred for confirmation. We postulate that this discordance between the SFLC ratio and SPE may be explained by the tendency of λ FLC to form dimers, which could obscure target epitopes necessary for detection by the SFLC assay antibodies. As described in a previous study, the SFLC assay had a relatively high false-negative rate compared to SPE and IFE in identifying patients with a λ M-protein.^20^ In the presence of an inflammatory disease process, it would then be possible to see a mildly increased ratio.

It is important to note that an abnormal SFLC ratio is not entirely specific to the presence of a MG. With decreases in renal glomerular filtration rate, there is a corresponding decrease in renal clearance of FLC, leading to a mild increase in the SFLC ratio in the absence of MG.^13^ A large number of patients with an abnormal SFLC ratio and no M-protein on SPE in our study cohort could be explained by renal impairment. Applying the proposed renal reference interval would then allow them to be classified as having a normal SFLC ratio. It is not uncommon for myeloma patients to present with renal impairment; one patient with renal failure and absent M-protein on SPE was determined to have MM on bone marrow biopsy. In this case, the ratio was 0.13, falling outside of the renal reference interval (0.37–3.1).^13^ In addition to renal impairment, inflammatory disorders such as autoimmune diseases have been identified as causes for elevated SFLC concentrations and a normal to mildly raised κ/λ ratio due to polyclonal increases in light chain production.^21,22^ In our study, many patients with normal renal function, abnormal SFLC ratio and absent M-protein had a polyclonal increase in gamma globulins on SPE. This could imply the presence of an inflammatory process. Most of these requests were from sub-specialities of Internal Medicine, in particular from pulmonology, neurology and rheumatology. Other studies have reported on the finding of a mildly raised κ/λ ratio with inflammatory disorders, and alternative reference intervals with higher upper limits have been suggested to reduce the rates of false positive ratios while maintaining diagnostic sensitivity. Hill et al. (2006) showed that the positive predictive value for an increased ratio improved from 34% to 58% when a cut-off of > 3.0 was used, and further improvement to 78% when a cut-off of > 5.0 was applied.^23^ Similarly, Sandfeld-Paulsen et al. (2022) found that increasing the ratio cut-off to 4.32 allowed a reduction in false positive results from 19% to 6%.^24^

As noted in previous guidelines, SFLCs are especially useful in identifying and monitoring light-chain MM and light-chain amyloidosis.^3,10^ In the current study, four patients with negative SPE and abnormal ratios had the diagnoses of light-chain MM and solitary plasmacytoma. This finding demonstrates the utility of requesting both SPE and SFLC in the initial screening of patients. Studies have shown that this combination of tests allows identification of virtually all cases of MG,^25,26^ although UPE and UIFE is still recommended when screening for light-chain amyloidosis.^10^

The current study focused on new patients not previously identified with a MG as there is ongoing discussion regarding the ideal MG screening test combination: SPE/IFE and SFLC or SPE/IFE and UPE/UIFE.^1,27^ In a recently published clinical update article, Rajkumar recommended that the initial investigation for a patient suspected of having MM should include SPE, serum IFE and SFLC.^1^ This is based on a study by Katzmann et al., who identified 428 patients with positive M-protein on urine IFE and determined the diagnostic sensitivity of different screening strategies. They determined that SPE with IFE alone would have missed 6.5% of patients (primarily light chain amyloidosis), while SFLC alone would have missed 14% of patients. The combination of SPE, IFE and SFLC identified 99.5% of patients. The two cases missed by this combination were diagnosed with MGUS and did not require medical intervention.^25^ In practice, collecting a single serum specimen rather than collecting both serum and urine from patients for screening purposes is also more convenient.

In a review article, Singh described SPE and UPE with IFE as the gold standard for diagnosing MG.^27^ When a M-protein is identified on UPE, it is diagnostic of MG, while an abnormal SFLC ratio is not. Therefore, the test combination of SPE/IFE and UPE/UIFE does not produce false positive results as with SFLC testing.^27^ False positive results may lead to unnecessary further investigation and patient anxiety.^28^ While the above is true, it is crucial to consider that UPE is typically a semi-automated method and, therefore, more labour-intensive than fully automated SFLC analysis. Automation allows higher throughput and shorter turnaround time for SFLC testing. Urine protein electrophoresis and UIFE also require visual inspection and manual interpretation, leaving room for human error.

### Limitations

A limitation of our study is the small number of patients included in the comparison of κ/λ ratio with SPE. Samples for SFLC analysis are received from multiple sites around the country; however, only a limited number of these sites use electronic medical record-keeping. It was not feasible to trace the physical medical records of these patients, and we were, therefore, limited to patients assessed at our centre. A further limitation is that we did not determine the diagnostic sensitivity and specificity of the available screening strategies due to the small numbers of patients with biopsy-confirmed MM included in the study. Due to the study’s retrospective nature, we did not look at other markers of inflammation, which may be helpful in patients with a mildly increased κ/λ ratio and absent M-protein on SPE.

### Conclusion

This study is one of the first to report on SFLC findings in an African context. It highlights challenges unique to a lower- to middle-income setting, such as the high rates of patients lost to follow-up or incomplete investigation and low volumes of tests received from primary and secondary care levels.

The authors have also demonstrated some of the strengths and limitations of SFLC and SPE methods in screening patients for MG. The findings showed that many SFLC results were in keeping with an inflammatory pattern, and hence, in patients suspected to have MG, SFLC requests should be based on a high index of clinical suspicion and performed with SPE. As per the literature, the most critical finding is that neither SFLC nor SPE should be performed in isolation to ensure no patient is missed.
